# Numerical Simulation of Vehicle–Lighting Pole Crash Tests: Parametric Study of Factors Influencing Predicted Occupant Safety Levels

**DOI:** 10.3390/ma14112822

**Published:** 2021-05-25

**Authors:** Paweł Baranowski, Krzysztof Damaziak

**Affiliations:** Institute of Mechanics & Computational Engineering, Faculty of Mechanical Engineering, Military University of Technology, 2 Gen. S. Kaliskiego Street, 00-908 Warsaw, Poland; krzysztof.damaziak@wat.edu.pl

**Keywords:** crash test, impact severity index, SPH, soil, vehicle, FEA

## Abstract

In this paper, numerical simulations of the EN 12767 test procedure for a vehicle–lighting pole crash are presented. A representative soil–vehicle–lighting pole model is first developed. The Geo Metro vehicle model is used, and significant attention is given to representing the soil and its interaction with the traffic pole. Soil is represented using smoothed particle hydrodynamics (SPH) coupled with finite elements (FEs). A parametric study is carried out to investigate the key factors influencing the outcomes and consequently the estimation of the occupant safety levels during crash scenario described in EN 12767. First, a sensitivity study of lighting pole mesh is conducted As a result, the optimal mesh size is used for further studies regarding physical parameters such as soil properties and friction coefficient in vehicle–pole interfaces. Friction and mesh size are found to have a considerable influence on the acceleration severity index (ASI), theoretical head impact velocity (THIV), post-impact velocity and vehicle behavior during the lighting pole crash scenario.

## 1. Introduction

Mitigating the negative consequences of vehicle collisions with roadside hardware is a major area of research in civil and automotive engineering. Roadside hardware includes barriers, lighting poles, closed-circuit television camera towers, overhead power transmission lines and security towers. Poles or posts made from steel or concrete are widely used in highway and street lighting systems and in structural applications. In 1998, the European Parliament proposed that primary action should be taken to halve fatalities due to roadside hardware collisions by 2010. In response, standard EN 12767 [1], approved by CEN (Comité Européen de Normalisation), was established in 2007 to define safety categories for support structures and direct rules for crash testing and interpreting the results.

According to EN 12767, road equipment support structures are classified based on the occupant safety levels which are described by the maximum values of the acceleration severity index (ASI) and the theoretical head impact velocity (THIV). Although the reliability of ASI and THIV as injury metrics describing the potential of impact to cause vehicle occupant injury has been questioned [2,3,4], these indexes remain the industry standards for measuring the safety level of road infrastructure. Pole certification is based on experimental passive safety tests. However, road infrastructure manufacturers are seeking to reduce the number of these tests by implementing numerical simulations. While numerical methods have proved highly efficient for modeling and simulating problems related to road traffic accidents and passenger safety [5,6,7,8,9,10], standards usually do not include detailed requirements on modeling techniques. A review of the literature also indicates great variation in approaches to crash test simulation. Despite the significance of the problem of passenger safety in vehicle collisions with roadside obstacles, few vehicle–pole crash test analyses have been reported. For example, [11] examines the influence of yield stress and thickness on pole behavior during a crash using a very simple vehicle model. An even simpler vehicle model is used in [12] to investigate the pole’s response to varying speed and mass of the impacting vehicle. In both studies, artificial boundary conditions were applied for the pole bottom. In [13], the authors used a simplified vehicle model but focused on a realistic representation of the concrete barrier. Spring elements representing the soil surrounding a pole were used in [14] to investigate the influence of different pole supports on crash parameters.

The above investigations adopted a simplified approach to simulating crash tests. At the other end of the modeling methodology spectrum are investigations featuring very detailed representations of a vehicle and pole. In [15], the influence of the vehicle model on the resulting ASI values was investigated. An equally detailed vehicle model was used to optimize the structure of a composite pole in [16]. By contrast, a steel pole was experimentally and numerically investigated in crash tests according to EN 12767 in [17]. These studies all focus on the application aspects of the simulation results obtained and generally omit validation and verification of the numerical models.

The status of numerical modeling is somewhat different for guardrails [13,18,19,20]. Utilization of numerical modeling of guardrail impact began in the 1980s with the development of computer programs such as GUARD and NARD [21]. A decade later, [21,22] and others discussed the quality and accuracy of finite element (FE) models of road hardware, focusing mainly on guardrails, and problems of accuracy and proper modeling techniques have since appeared regularly in the literature. Ref. [23] presented a roadmap for proper modeling of guardrails. In 2011, a book describing the validation process in detail was published [24]. Ref. [25] presented a methodology for validating and verifying numerical models using uniaxial tensile test data and the results of frontal impact tests using the sledge stand. Ref. [26] focused on improving the fidelity of numerical representations of the wheels of a vehicle impacting a guardrail, and [27] discussed the set of elastoplastic parameters of a chosen constitutive model of soil that will yield the best correlation of the calculation results with experimental results.

The present paper focuses on numerical simulations of the EN 12767 test procedure for the vehicle–lighting pole crash interaction. The main aim of the study is to highlight the importance of considering several factors influencing predicted occupant safety levels. Accordingly, a parametric study with a series of numerical simulations is carried out to investigate the key factors that can have an impact on the outcomes and, consequently, the reliability of finite element analysis (FEA) in crash test scenarios. In the first step, the influence of mesh size on the results was investigated. Based on the results, the optimal size of FE mesh was selected for further studies concerning physical parameters of the model, i.e., soil and friction properties in the vehicle–lighting pole interface. Additionally, the developed model is validated with the experimental and numerical observations of a similar lighting pole studied in [17]. The Geo Metro vehicle model and a representative model of a traffic pole are adopted. Significant attention is given to representing the soil and its interaction with the traffic pole. The results demonstrate the need to verify the model with respect to the analyzed factors before it can be adopted for reliable simulation of a crash test scenario with a traffic pole.

The arbitrary choice of investigated parameters was driven mainly by the literature review presented above. The author’s experience in the field of practical, industrial simulations also supports this selection. These parameters are often taken from the literature (friction coefficients and soil parameters) or are based on the “best practice” (mesh size), and in most of the refences are not investigated properly. On the other hand, they can lead to misleading judgements of the level of human safety during a collision with a lighting pole. Obviously, there are many other aspects influencing the results of numerical simulations including the formulation of finite elements, number of integration points or hourglassing control (in case of under integrated elements) [28]. Nonetheless, the three parameters investigated in this study can be considered as potential sources of misleading outcomes of simulations of the crash scenario described in the EN 12767.

## 2. Occupant Safety Levels Description

The parameters used for classification of the equipment support structures according to EN 12767 are the velocity of the vehicle’s center of gravity (CG), energy absorption and passenger safety. The energy absorption category of the pole is estimated by measuring the post-impact velocity of the vehicle after a covered distance of 12 m after the impact, as outlined in Table 1. Pole certification is based on experimental passive safety tests. As shown in Table 2, one crash test is performed at a vehicle speed of 35 km/h to assess how the pole will interact with the vehicle at low velocity, and then another test is performed at a higher velocity (50, 70 or 100 km/h) depending on a desired passive safety classification. Thus, a performance type of the tested support structure is expressed in the format 100HE3 (speed class, energy absorption category and occupant safety level). It means that for a vehicle impacting at 100 km/h, the post-impact speed is between 0 and 50 km/h, and the maximum ASI and THIV values are less than 1.0 and 27 km/h, respectively.

Based on the measurements during testing, ASI and THIV parameters are determined. The ASI and THIV values should be smaller than the maximum limits shown in Table 2. The ASI is a dimensionless measure of the acceleration of the vehicle’s CG. It describes the intensity of deceleration acting on the occupant at the time of collision with an obstacle [1,29]:

(1) $$ASI(t)=\sqrt{{\left(\frac{{\bar{a}}_{x}(t)}{12g}\right)}^{2}+{\left(\frac{{\bar{a}}_{y}(t)}{9g}\right)}^{2}+{\left(\frac{{\bar{a}}_{z}(t)}{10g}\right)}^{2}},$$

where ${\bar{a}}_{x},\;{\bar{a}}_{y},\;{\bar{a}}_{z}$ are the time-dependent components of accelecration along the *x, y* and *z* axes recorded at vehicle’s CG and *g* is the acceleration due to gravity.

THIV is the velocity, expressed in (km/h), at which a hypothetical occupant’s head (represented by a point mass) hits the surface of the hypothetical vehicle’s interior. The value is calculated according to the procedure defined in the norm [29]. The trajectory of the hypothetical head and THIV are calculated from the parameters measured in the determination of the vehicle’s CG. A general equation describing THIV is as follows:

(2) $$THIV=\sqrt{V_{x}{}^{2}(T)+V_{y}{\left(T\right)}^{2}},$$

where $V_{x},\;V_{y}$ are the *x* and *y* components of theoretical velocity of the head with respect to the vehicle.

## 3. Problem Description

### 3.1. Model Definition

**Figure 1:**
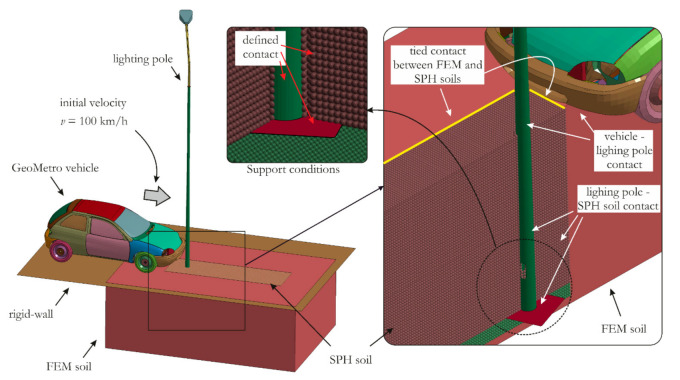
The FE model of the vehicle–soil–lighting pole system with initial–boundary conditions. Inset: close-up view of soil modelled using SPH particles (for better visibility, the soil is cut in half).

To simulate the crash scenario, a numerical model was developed consisting of soil, a vehicle and a lighting pole. The assumptions of all simulations were as follows:

- FEA was conducted with implementation of massive parallel processing (MPP) LS-Dyna R10.1.0 explicit code.
- The soil was modeled using constant stress solid FEs with one integration point and with stiffness-based hourglass control. The average FE size was 30.0 mm. The elements were coupled with smoothed particle hydrodynamics (SPH) particles [30,31,32,33,34], which were used to represent the area within direct interaction with the lighting pole. The soil area in the present study was larger than the requirements presented in [1], with dimensions of 3.6 m, 5.3 m and 1.9 m for width, length and height, respectively. This choice was made due to the possible large deformation of the soil during lighting pole deflection depending on the constitutive model used. To couple the SPH particles within the area of direct interaction with the lighting pole, a kinematic constraint method in which the particles are tied to the Lagrangian surface was applied in order to maintain the continuity of the soil. The outer surfaces of the soil area were fixed.
- The SPH soil was modelled using the renormalization approximation, which is recommended for most applications [35]. A sensitivity study of particle density is not presented, since a very small influence on the results was observed. Ultimately, a regular grid of particles was used with a space between particles equal to 20.0 mm. Moreover, the recommended artificial bulk viscosity coefficients *Q*_1_ = 1.0 and *Q*_2_ = 1.0 were used for the SPH soil [35].
- The representative traffic pole with a height of 8.0 m and a diameter of 142.0 mm and 56.0 mm in the bottom and in the upper part of the pole, respectively, was adopted. The pole was mainly made of steel and was mounted into the ground at a depth of 1.6 m. To represent the ground–lighting pole interaction, a contact between the pole column and bottom plate was used. For the sake of a better presentation, the SPH soil was divided into two parts located above and below the bottom plate (Figure 1). The pole was discretized using fully integrated Lagrangian Belytschko-Tsay (BT) shell elements.
- For the vehicle, the widely used and deeply validated Suzuki Geo Metro FE model was adopted [20,25,26,27] as modified by the Department of Mechanics of Materials and Structure, Gdańsk University of Technology, Poland [26]. The model consists of 14,709 shell elements and 820 solids. Additionally, spring and discrete dampers are used to model shock absorbers. The majority of the parts in the model are modelled using piecewise linear plasticity material model with erosion criteria.
- The interactions between all parts in the model were simulated using a penalty function approach adopting Coulomb’s friction model [36,37,38]. In addition to friction properties between vehicle and lighting pole, the Coulomb’s friction coefficients of *μ =* 0.1 and *μ =* 0.4 for steel–steel and tire–ground pairs were used in the model, respectively.
- Numerical simulations were carried out with a vehicle velocity of 100 km/h; the outcomes were analyzed considering the energy absorption category HE and an occupant safety level of 3 (see Table 1 and Table 2).
- To validate the model, the numerical simulations were compared with observations presented in [17], where similar testing conditions were presented.

The above-defined model, which was considered in all simulations, is illustrated in Figure 1, where initial-boundary conditions are also highlighted. The overall numbers of elements and nodes representing the whole model were 468,523 (including 378,358 SPH particles) and 479,475, respectively. Obviously, the numbers of elements and nodes varied depending on the mesh size used to represent the lighting pole, and the given model data are for a shell element size of 15 mm.

### 3.2. Constitutive Modeling

Since the simulated crash scenario is a dynamic phenomenon in which the strain rate effect plays a significant role, the widely used Johnson–Cook (JC) constitutive model [39,40,41,42,43] was implemented to model the behavior of the lighting pole. Specifically, the simplified version of the JC model (SJC) was adopted. The SJC model provides predictions of flow stress *σ_flow_* for large strains and high strain rates when its dependence on the strain rate is linear on a semi-logarithmic scale [35]:

(3) $$\sigma_{flow}=\left[A_{JC}+B_{JC}{\left(\varepsilon^{p}\right)}^{n}\right]\left(1+C_{JC}\ln{\dot{\varepsilon }}_{*}^{p}\right),$$

where *A_JC_, B_JC_, C_JC_, n* are material constants, $\varepsilon^{p}$ is the effective plastic strain and ${\dot{\varepsilon }}_{*}^{p}$ is the effective plastic strain rate.

A steel sheet thickness for pole fabrication of 2.0 mm was considered, and thus the parameters for the SJC model were based on [44], where a S235 steel sheet of the same thickness was experimentally tested and simulated. The parameters necessary to apply in the SJC model card in LS-Dyna are listed in Table 3.

The soil was simulated using the soil and foam (SF) constitutive model with the parameters determined for three different types of cohesive soils, namely S, M and H [27]. The parameters for the SH model for each cohesive soil were taken from [27], where a detailed description of the model and its parameters can be also found. The SF model is sufficient to model the dynamic behavior of soils subjected to various types of loading [15,27,45,46]. The SF model requires the following parameters: bulk modulus for unloading (*BULK*), shear modulus (*G*), yield function constants (*a*_0_, *a*_1_, *a*_2_), pressure cut-off for tensile fracture (*PC*), and optionally pressure vs. volumetric strain curve. The SF model for soil uses the pressure-dependent nonlinear Drucker–Prager yield function $\phi_{DP}$, which is described in terms of the second invariant *I*_2_ of the deviatoric stress tensor *s_ij_*, pressure *p,* and constants *a*_0_, *a*_1_, *a*_2_ as follows [35]:

(4) $$\phi_{DP}=I_{2}=\frac{1}{2}s_{ij}s_{ij}=a_{0}+a_{1}p+a_{2}p^{2}$$

The three types of cohesive soils mentioned above were included in FEA because one of the aims of the present study is to analyze the influence of different types of soils. For a detailed description of the outcomes depending on soil properties, please see Section 5.2.2. The corresponding input parameters in the SF model LS-Dyna card for soft (S), medium (M) and hard plastic cohesive (H) soils are presented in Table 4.

## 4. Methodology of Numerical Simulations

### 4.1. Model Validation

In the mentioned study, a pole with a height of 12.0 m was used, made of S355 steel tube with a thickness of 2.0 mm. The experiments were conducted according to EN 12767 standard and 100HE3 safety class. The numerical simulations were also carried out and LS-Dyna code was utilized. The lighting pole was attached to a prefabricated concrete foundation, which was installed in soil. The Seat Ibiza car was used in experimental tests and the Geo Metro vehicle model was used in numerical simulations. Despite some differences compared to the present paper, the outcomes from [17] were considered as representative enough for comparison with the results presented here. To compare the results, a model with the same element size (10.0 mm) used for lighting pole discretization as in the reference paper was chosen. The present and referenced studies are summarized in Table 5 where common and different elements of both investigations can be distinguished.

### 4.2. Description of Crash Test Scenarios

**Figure 2:**
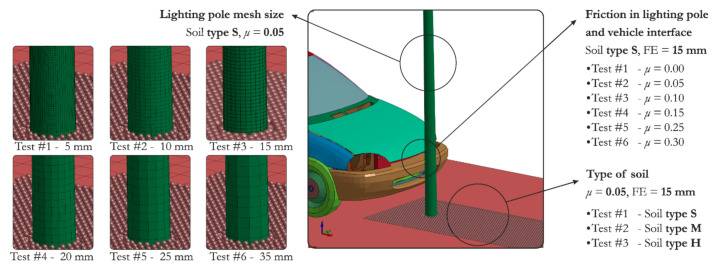
Description of test conditions adopted in the simulations in the present study.

The factors considered in this study were analyzed in three stages. First, the optimal size of FE mesh was selected. Next, further studies were conducted concerning physical parameters of the model, i.e., soil and friction properties in the vehicle–lighting pole interface (Figure 2). All simulated cases are summarized in detail in Table 6. Although two coefficients of friction (*μ_s_* and *μ_d_*) can be distinguished, the Coulomb’s friction coefficient *μ* is used throughout the paper. In fact, during such dynamic vehicle–pole interaction *μ* equals to *μ_d_* nearly for a whole duration of vehicle and lighting pole interaction.

#### 4.2.1. Mesh Size

A mesh parametric study was conducted, in contrast to the simplified model of a vehicle adopted in a similar study [12]. Mesh properties can drastically influence the response of a discretized structure, and these effects are more pronounced in simulations of shell structures [28]. Obviously, when a finer mesh is used, a decrease in numerical error is expected to occur. Upon impact of the vehicle, a large deflection of the lighting pole is observed, and thus it can be assumed that the size of the mesh will also be of great importance in this scenario. The results of FEA in Section 5.2.1 confirm the influence of mesh size. The lighting pole was discretized using selected sizes of mesh ranging from fine (5 mm) to coarse (35 mm) and the soil was simulated using the SF material model with the parameters of soil type S. The Coulomb’s friction coefficient was taken as *μ* = 0.05.

#### 4.2.2. Soil Properties

The EN 12767 standard provides only brief, basic data on the soil that should be used in certification tests (the S type of cohesive soil). Nevertheless, studies of vehicle–lighting pole crash test simulations report various data of soils used in numerical simulations [14,15,46]. Other papers do not describe the soil parameters used in analysis [16,47]. Despite the large number of crash test investigations, papers dealing with soil properties and their influence on vehicle behavior during a crash test scenario are scarce, particularly for vehicle–lighting pole impact simulations. The few relevant papers modeled soil using spring elements to investigate the influence of soil properties on crash parameters [14,48]. The soil type can affect vehicle behavior, and special attention is needed when adopting soil parameters, as outlined in Section 3.1. To analyze the influence of soil type on the outcomes, three different soils with the properties shown in Table 4 were considered. The EN 12767 standard includes the S type of soil, and thus the choice of soils with such different properties may at first glance seem inappropriate. However, in many studies of crash test simulations, the soil data used are uncertain, making it difficult to judge whether the selected soil meets the requirements of the EN 12767 standard. Thus, in Section 5.2.2, changes in vehicle behavior depending on soil type are presented.

#### 4.2.3. Friction Properties

Previous studies dealing with friction properties and/or their impact on the obtained results are subject to two distinct limitations [18,23,25,49,50,51,52,53]. First, the friction data are not consistent, and significantly different values of friction coefficients are assumed despite the simulation of similar problems. Second, friction properties are mentioned only in studies of safety road barrier crash tests. In [49], the authors used a Coulomb’s friction coefficient of *µ =* 0.25 for vehicle parts vs. steel barrier parts. By contrast, in [18], contact between the barrier and the vehicle was simulated using *μ_s_* = 0.42 and *μ_d_* = 0.10. Frictionless contact between the vehicle and guardrail was defined in [52], whereas *μ_s_* = 1.0 and *μ_d_* = 0.2 were chosen in [51] to model contact between the vehicle and barrier as well as between the vehicle and asphalt pavement. Furthermore, in the LS-Dyna manual, typical values of friction for hard steel vs. hard steel and mild steel vs. mild steel are given as *μ_s_* = 0.78 and *μ_d_* = 0.08 (greasy), 0.42 (dry) and *μ_s_* = 0.74 and *μ_d_* = 0.10 (greasy) and 0.57 (dry), respectively. Since the behavior of a vehicle is strongly affected by friction [54], as discussed in Section 5.2.3, the credibility of numerical modeling is questionable when friction data are not included. Therefore, in the last stage of the investigation, different properties of friction were assumed for the vehicle–lighting pole interface. The mesh size (15 mm) and soil properties (S type) remained unchanged for all simulated cases.

## 5. Results and Discussion

### 5.1. Model Validation

**Figure 3:**
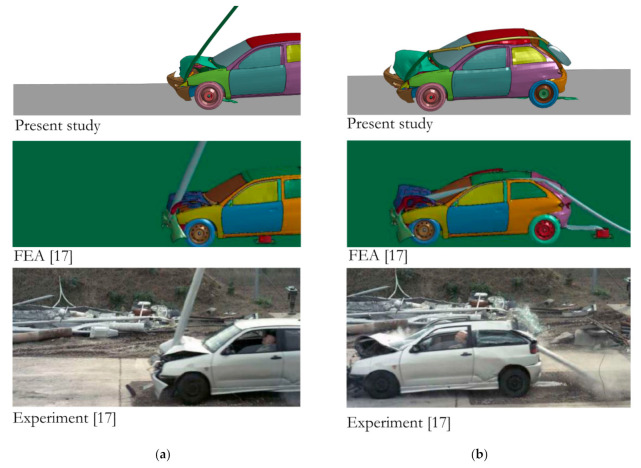
Vehicle behavior during impact with the lighting pole: comparison between the present study and results from Ref. [17]; (**a**) t = 0.1 s, (**b**) t = 0.25 s.

Prior to the parametric study, the model was validated with the outcomes from the similar tests presented in [17]. In Figure 3, a vehicle impacting a lighting pole for the selected moments of time is presented; the results obtained from the numerical simulations of the present study and from the experimental tests with numerical simulations from [17] are shown.

A similar behavior of the vehicle and lighting pole can be observed compared to the reference outcomes. A significant deformation of the lighting pole occurs due to impact, which consequently causes the pole to wrap around the vehicle body. Naturally, due to the different pole heights, some differences in the pole–vehicle roof interaction are apparent. Nevertheless, the results are consistent with the observations reported in other works on this subject [15,16,46,47]. According to [17], maximum values of ASI and THIV were close to 1.0 and 30 km/h, respectively. In the present paper, the obtained maximum values of ASI and THIV were 0.79 and 31.39 km/h, respectively. The discrepancies are due to differences between the present paper and Ref. [17], which are also mentioned in Section 4.1.

### 5.2. Parametric Study

To assess the influence of selected factors on vehicle behavior, the following parameters were analyzed:

- history of velocity measured for the center of mass of the vehicle;
- history of ASI;
- vehicle behavior during crash impact;
- overall deformation of the lighting pole and soil;
- maximum values of ASI and THIV and minimum values of velocity.

#### 5.2.1. Influence of Mesh Size

The accuracy of the solution obtained by FE analysis depends on the mesh size. A coarse mesh results in larger calculation errors, while increasing the number of elements minimizes the error and asymptotically approaches the exact solution [55,56]. Furthermore, the size of the element used to develop a discrete model can drastically change its stiffness and resistance. In the case of crash analysis, where large deformations of a vehicle body are inevitable, another aspect of discretization must be considered. The size of the elements, especially in the crumple zones, should permit a proper description of the vehicle’s structure. The use of FEs that are too large will prevent a proper description of the crumpling phenomenon, resulting in overly stiff behavior of vehicle body and improper energy balance caused by insufficient transformation of energy from kinematic to internal.

**Figure 4:**
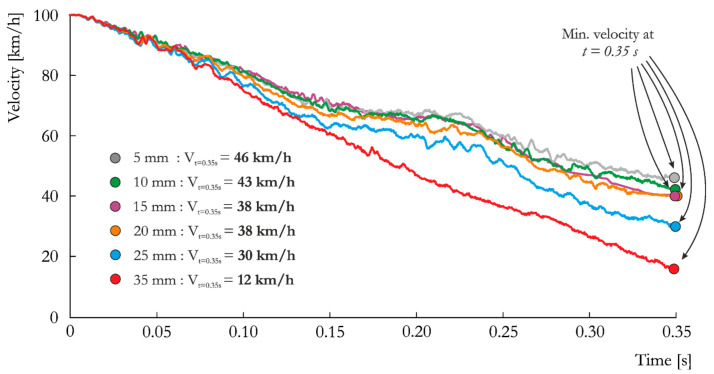
Vehicle velocity vs. time curves obtained for the analyzed mesh sizes: 5 mm, 10 mm, 15 mm, 20 mm, 25 mm and 35 mm.

The above-mentioned issues of discretization of the problem are reflected in the analyses performed here. Figure 4 shows the change in vehicle velocity over time for the analyzed mesh sizes. A coarser mesh obviously tends to result in a smaller value of vehicle velocity at *t* = 0.35 s. The differences are relatively small for the curves representing element sizes of 5 mm to 20 mm. However, for the S_25_0.05 case, significant deceleration of the vehicle is observed, and for the coarser mesh with a size of 35 mm, the velocity is equal to *v =* 12 km/h, which is approximately four times smaller than the minimum velocity measured for the 5 mm mesh. This is mainly attributable to vehicle pitching, which is discussed later in the paper.

**Figure 5:**
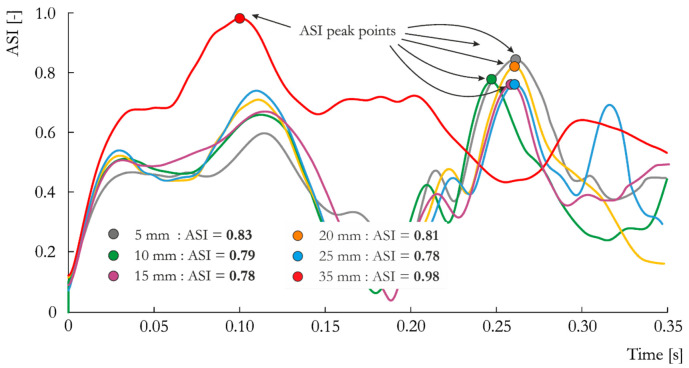
ASI vs. time curves obtained for the analyzed mesh sizes: 5 mm, 10 mm, 15 mm, 20 mm, 25 mm and 35 mm.

A general trend in the velocity histories associated with the change in element size is visible at the first peak of the ASI vs. time curves presented in Figure 5, i.e., at approximately *t =* 0.1s. In general, the ASI value increases with increasing element size due to the change in local stiffness of the vehicle model in the area of contact with the lighting pole, which directly influences the description of local crumpling of the vehicle body. Despite this visible trend in the first peak of the curves, the maximum values of ASI for all simulated cases, expect S_35_0.05, occurred in the last peak. The maximum ASI for the element size of 35 mm is a result of vehicle pitching (discussed later in the paper). It is worth mentioning that for all mesh sizes, the lighting pole fulfills the EN 12767 standard requirements according to the post-impact velocity and ASI (Table 1 and Table 7).

The 15 mm mesh was able to represent the problem without a visible influence on car behavior. Therefore, it was further used for studies concerning physical parameters of the model, i.e., soil and friction properties.

#### 5.2.2. Influence of Soil Type

**Figure 6:**
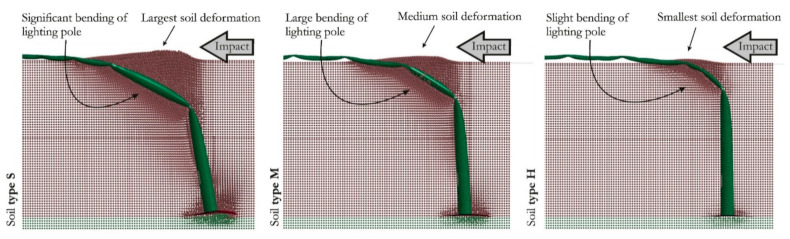
Soil type interaction at the maximum deflection of the lighting pole—comparison between three analyzed types of soil.

In this stage, the mesh size of 15 mm and Coulomb’s friction coefficient *μ* = 0.05 were considered. The influence of soil type on vehicle behavior manifests via the soil–pole interaction. The harder the soil, the more difficult it is to move the foundation part of the pole, which changes the overall stiffness characteristic of the pole as an obstacle. The correlation between soil hardness and vehicle behavior is clearly evident in the results. The resistance of the pole set in hard soil is more pronounced than the resistance of the pole set in soft soil (Figure 6).

**Figure 7:**
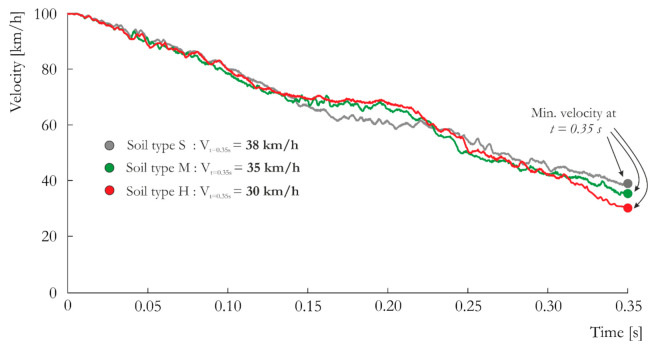
Vehicle velocity vs. time curves obtained for the three analyzed types of soil: S, M and H.

The observed differences in lighting pole behavior during impact among the three tested types of soils directly influence the change in vehicle velocity (Figure 7) Due to significant deflection of the pole in soft soil, sudden deceleration of the vehicle is not observed. By contrast, among the three soil types, deceleration during impact is greatest when the pole is set in the hard, plastic cohesive soil, and the velocity after impact is lowest (38 km/h compared to 32 km/h). Ultimately, the lighting pole fulfills the EN 12767 standard requirements according to the post-impact velocity, which should be smaller than *Ve =* 50 km/h (Table 1 and Table 7).

**Figure 8:**
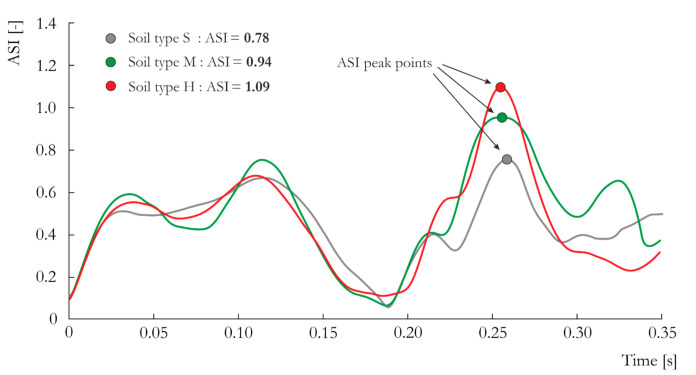
ASI vs. time curves obtained for the three analyzed types of soil: S, M and H.

The observations of soil–lighting pole behavior and velocity histories are confirmed by the ASI histories. In Figure 8, the ASI vs. time characteristics are presented for the three analyzed soils. The influence of soil type is apparent throughout all parts of the curve. For the soft cohesive soil, approximately 20% smaller value of ASI was obtained (0.78) compared to the soil type H (1.09). This point corresponds to the moment just after the initial contact of the vehicle with the pole and the initial crumpling of the front of the vehicle body. After this stage, the pole starts to deform considerably, and its foundation interacts with the soil, preventing the pole from being torn out of the ground. It is worth noticing that the value of ASI = 1.09 means that the tested lighting pole does not meet the requirements of the EN 12767 standard.

#### 5.2.3. Influence of Friction

**Figure 9:**
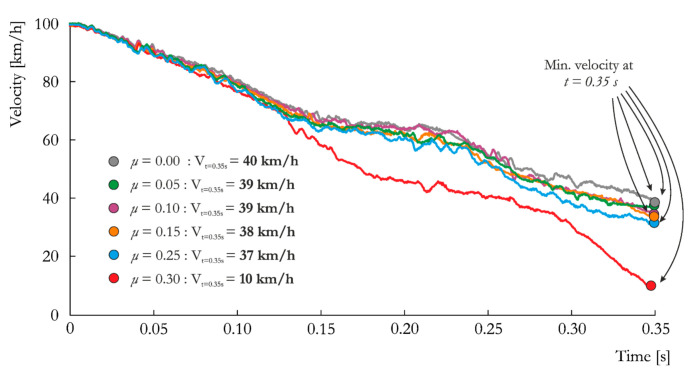
Vehicle velocity vs. time curves obtained for the analyzed friction coefficients: *μ* = 0.00, *μ* = 0.05, *μ* = 0.10, *μ* = 0.15, *μ* = 0.25 and *μ* = 0.30.

In the last stage, the mesh size of 15 mm and soil type S were considered. The influence of the friction coefficient on vehicle deceleration is presented in Figure 9. As expected, increasing the friction coefficient results in a larger decrease in velocity over time. However, this decrease in velocity is less pronounced for S_15_0.00 through S_15_0.25. Therefore, friction coefficient values between 0.0 and 0.25 do not affect the results significantly, and all curves are approximately the same. Starting from *μ* = 0.25, the influence of friction on the results becomes more evident, and vehicle behavior changes completely at *μ* = 0.30 (discussed later in Section 5.3)). Notably, for this last case, S_15_0.30, the measured velocity is smallest among all analyzed cases with different mesh sizes and soil types, as presented in Figure 4 and Figure 7, respectively. For all cases, the measured velocity values are between the range defined in the EN 12767 standard (Table 1 and Table 7).

**Figure 10:**
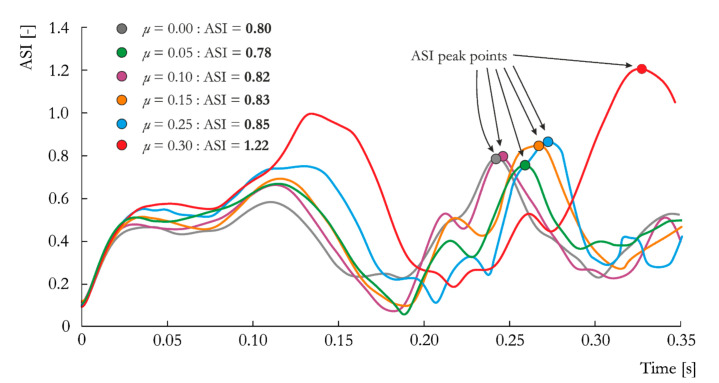
ASI vs. time curves obtained for the analyzed friction coefficients: *μ* = 0.00, *μ* = 0.05, *μ* = 0.10, *μ* = 0.15, *μ* = 0.25 and *μ* = 0.30.

The ASI histories for the cases with different friction coefficients are presented in Figure 10. Like the case of different mesh sizes, not the highest values but the time point of *t* = 0.12 s warrants attention. A trend of the influence of the friction coefficient is clearly evident at this time point. The maximum ASI values occur at the peaks at *t* = 0.20–0.35 s and, again, are quite similar for all cases but *μ* = 0.30. The ASI values are within the range of 0.78–0.85, whereas for the last case, the ASI value is far above 1.0, with a value of 1.22. Assuming that the tested lighting pole meets the requirements of the EN 12767 standard with an occupant safety level of 100HE3, the overall verdict of the numerical analysis changes from “passed” for, e.g., *μ* = 0.10, to “failed” for *μ* = 0.30. Furthermore, adopting the typical values of friction for hard steel taken from the LS-Dyna manual would yield significantly different results.

### 5.3. Comparisons between Crash Test Scenarios

**Figure 11:**
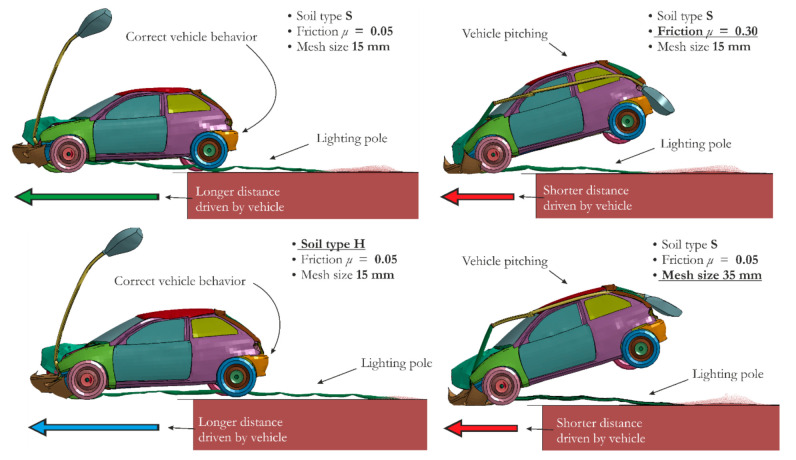
Vehicle behavior during impact with the lighting pole at t = 0.35 s: comparison between the cases with the most critical analyzed parameters, i.e., soil type H, friction coefficient μ = 0.30 and mesh size of 35 mm.

To determine which factor has the largest impact on the outcomes, the values of ASI, THIV and velocity at *t* = 0.35 s are compared and presented in Table 7. Alongside each value of the three criteria, the (+) and (−) symbols are also included to show which cases “passed” or “failed” the requirements of EN 12767 standard. Furthermore, Figure 11 compares vehicle behavior during impact with the lighting pole at *t* = 0.35 s for the most critical analyzed parameters (factors): mesh size of 35 mm, soil type H and friction coefficient *μ* = 0.30.

In the mesh sensitivity study, reducing the element size by seven-fold results in a decrease in the ASI value from 0.98 to 0.83 (a difference of ~15%). For the finest mesh, THIV is 29.82, whereas for S_35_0.05, a value of 36.55 is obtained. Thus, a finer mesh size results in approximately 20% lower THIV compared with the coarsest mesh used in the simulations. Among all cases, the largest velocity is observed for S_05_0.05, at t = 0.35 s. By comparison, when a mesh size of 35 mm is used, the velocity is almost four times smaller. Furthermore, the change in element size affects the overall behavior of the vehicle during impact. Both the parameters and vehicle behavior illustrated in Table 7 and Figure 11, respectively, indicate that using an FE size larger than 35 mm leads to very erroneous results.

The outcomes of the discussed cases show that changing the soil parameters affects the results quite significantly. The shear modulus differs by 10-fold between soil type S and soil type H, directly resulting in approximately 20% smaller values of ASI and THIV for the soft cohesive soil. Moreover, the velocity measured at *t* = 0.35 s is 21% greater for H_15_0.05 compared with S_15_0.05. Despite a considerable impact of soil type on safety parameters and velocity values, reasonable vehicle behavior without pitching is observed for all types of soil.

The friction coefficient has the most significant influence on the observed outcomes. Changing the friction coefficient from *μ* = 0.05 to *μ* = 0.3 results in a change in the THIV value from 31.59 to 36.78. Moreover, the smallest velocity among all analyzed cases, i.e., *V* = 10 km/h, is observed at the largest friction coefficient, as shown in Table 7. Friction coefficients equal to or greater than 0.30 lead to vehicle pitching (Figure 11), which does not actually occur in a real-world scenario.

## 6. Conclusions

Simulation programs based on FEM have become very accessible, including pre/post-processors that enable simple preparation of numerical models. As a result, even numerically complicated and difficult simulations can be prepared and performed by users with little experience in numerical modeling. Therefore, especially in the case of advanced simulations, discussions of the adopted FE modeling techniques and model sensitivity studies are necessary, as comparisons of numerical and experimental results can be insufficient to demonstrate whether the numerical model was correctly developed or not.

The present sensitivity study assessed the influence of FE mesh density, soil type and coefficient of friction in modeling EN 12767 crash tests. All three variables influenced the outcomes obtained, and in all three cases, sets of parameters changing the overall result of the simulation of test from “passed” to “failed” were identified. This is visible in the case of soil types S and H. On the other hand, a coarse mesh caused pitching of the vehicle, whereas a high friction coefficient caused both pitching and test failure due to a high ASI value.

Based on these results, it can be concluded that careless adoption of the above-mentioned parameters in simulations of a complicated phenomenon like the crash test defined in EN 1276 can easily lead to very misleading results, which ultimately have an impact on predicted occupant safety criteria. This somehow obvious conclusion follows the observation that most of the articles dealing with simulations of crashes against road infrastructure do not mention the verification stage in the numerical modelling process, nor explain the source of the parameters adopted in the models.

## Figures and Tables

**Table 1:** Energy absorption levels for support structure [1].

Impact Speed Vi (km/h)	Post-Impact Speed Ve (km/h) Measured at a Point 12 m beyond the Impact Point with the Support Structure		
	HE—Energy Absorption in the High Degree	LE—Energy Absorption in the Low Degree	NE—Non-Energy Absorption
50	Ve = 0	0 ≤ Ve ≤ 5	5 < Ve ≤ 50
70	0 ≤ Ve ≤ 5	5 < Ve ≤ 30	30 < Ve ≤ 70
100	0 ≤ Ve ≤ 50	50 < Ve ≤ 70	70 < Ve ≤ 10

**Table 2:** Occupant safety levels [1].

Energy Absorption Category	Safety Level of Occupant	Speed			
		Crash Test at a Speed of 35 km/hMaximum Values		Crash Test at a Speed of 50 km/h, 70 km/h or 100 km/hMaximum Values	
		ASI	THIV (km/h)	ASI	THIV (km/h)
HE	3	1.0	27	1.0	27
	2	1.0	27	1.2	33
	1	1.0	27	1.4	44
LE	3	1.0	27	1.0	27
	2	1.0	27	1.2	33
	1	1.0	27	1.4	44
NE	3	0.6	11	0.6	77
	2	1.0	27	1.0	27
	1	1.0	27	1.2	33

**Table 3:** Parameters for the SJC model adopted for the lighting pole [44].

Parameter	Variable	Unit	Value
Density	*ρ*	kg/m^3^	7850.0
Young’s Modulus	*E_JC_*	MPa	210,000
Poisson’s ratio	*v_JC_*	-	0.29
JC yield stress	*A_JC_*	MPa	235.0
Hardening parameter	*B_JC_*	MPa	520.0
Hardening parameter	*n*	-	0.638
Strain rate parameter	*C_JC_*	-	0.046
Failure strain	*Ps_fail_*	-	1.3

**Table 4:** Parameters for the SF material model adopted for the S, M and H soils [27].

			Value		
			Soil Type		
Parameter	Variable	Unit	S	M	H
Density	*ρ*	kg/m^3^	2100.0	2100.0	2100.0
Shear Modulus	*G*	MPa	2.75	10.0	27.5
Bulk modulus for unloading	*Bulk*	MPa	32.1	64.2	129.9
Yield function constants for plastic yield function	*a*_0_	MPa^2^	0.00058	0.0016	0.0024
	*a*_1_	MPa	0.010	0.019	0.037
	*a*_2_	-	0.045	0.078	0.140
Pressure cut-off fortension fracture (<0)	*PC*	MPa	−2.0	−2.0	−2.0
Bulk modulus for loading	*K*	MPa	10.7	21.4	43.4

**Table 5:** Comparison between the study [17] and the present paper.

Parameter	Referenced Study [17]	Present Study
Lighting pole height	12.0 m	8.0 m
Lighting pole thickness	2.0 mm	2.0 mm
Lighting pole material	S355 steel	S355 steel
Lighting pole mount type	concrete foundation	soil
Lighting pole mesh size	10.0 mm	10.0 mm
FEA code	LS-Dyna	LS-Dyna
Vehicle	Seat Ibiza (exp.) Geo Metro (sim.)	Geo Metro
Experiment type	EN 12767—100HE3	EN 12767—100HE3

**Table 6:** Test conditions with the three analyzed factors.

Analyzed Factor	Test Name	Factor Values in Each Test		
		Soil Type	Mesh Size	Friction Properties
Mesh	S_05_0.05	S	5 mm	*μ* = 0.05
	S_10_0.05		10 mm	
	S_15_0.05		15 mm	
	S_20_0.05		20 mm	
	S_25_0.05		25 mm	
	S_35_0.05		35 mm	
Soil	S_15_0.05	S	15 mm	*μ* = 0.05
	M_15_0.05	M		
	H_15_0.05	H		
Friction	S_15_0.00	S	15 mm	*μ* = 0.05
	S_15_0.05		10 mm	*μ* = 0.00
	S_15_0.10		15 mm	*μ* = 0.10
	S_15_0.15		20 mm	*μ* = 0.15
	S_15_0.25		25 mm	*μ* = 0.25
	S_15_0.30		35 mm	*μ* = 0.30

**Table 7:** Comparison of ASI, THIV and velocity for each tested case.

Test Name	Parameter		
	ASI (max. 1.0)	THIV (≤27 km/h)	Velocity (≤50 km/h)
**Mesh Size**			
S_05_0.05	0.83 +	29.82 −	46.0 +
S_10_0.05	0.79 +	31.39 −	43.0 +
S_15_0.05	0.78 +	31.59 −	38.0 +
S_20_0.05	0.81 +	32.18 −	38.0 +
S_25_0.05	0.78 +	32.85 −	30.0 +
S_35_0.05	0.98 +	36.55 −	12.0 +
**Soil Type**			
S_15_0.05	0.78 +	31.59 −	38.0 +
M_15_0.05	0.94 +	32.05 −	35.0 +
H_15_0.05	1.09 -	35.33 −	30.0 +
**Friction Coefficient**			
S_15_0.00	0.80 +	30.29 −	40.0 +
S_15_0.05	0.78 +	31.59 −	39.0 +
S_15_0.10	0.82 +	32.32 −	39.0 +
S_15_0.15	0.83 +	33.31 −	38.0 +
S_15_0.25	0.85 +	34.26 −	37.0 +
S_15_0.30	1.22 -	36.78 −	10.0 +

## Data Availability

The data presented in this study are openly available in Vibrations in Physical Systems at doi:10.21008/j.0860-6897.2020.3.07, reference number [17]; Engineering Transactions at doi:10.24423/EngTrans.1036.20190802, reference number [27]; International Journal of Impact Engineering at doi:10.1016/j.ijimpeng.2016.01.006, reference number [44].
